# Effects of Pig Weaning Weight and Sex on Growth Performance, Fecal Score, and Blood Antioxidant Capacity in Early Nursery Period

**DOI:** 10.3390/ani16142271

**Published:** 2026-07-22

**Authors:** Chan Ho Kwon, Ji Hye Lee, Yoon Soo Song, Shelby Greer, Eva S. Safaie, Jannell A. Torres, Young Dal Jang

**Affiliations:** 1 Department of Animal and Dairy Science, University of Georgia, Athens, GA 30602, USA; 2 Department of Animal Science and Technology, Chung-Ang University, Anseong-si 17546, Gyeonggi-do, Republic of Korea

**Keywords:** antioxidant status, growth, gut health, nursery pigs, sex, weaning weight

## Abstract

This experiment aimed to determine the effects of weaning weight (WW) and sex on growth performance, fecal score, gut permeability, and antioxidant status in pigs. At weaning (19.0 ± 1.35 d of age), 64 newly weaned pigs (32 barrows and 32 gilts) were allotted to two WW categories: high-WW pigs (>5.5 kg) and low-WW pigs (<5.5 kg) in eight replicates (four pens for barrows and four pens for gilts) for a 28 d feeding trial. High-WW pigs showed greater body weight (*p* < 0.01) than low-WW pigs every week. During the overall period, high-WW pigs showed greater weight gain (*p* < 0.01) and feed intake (tendency *p* = 0.06) than low-WW pigs. Weaning weight was positively correlated with overall weight gain (r = 0.853), feed intake (r = 0.714), and feed efficiency (r = 0.719) in gilts but there was no or negative correlation in barrows. Weaning weight showed a tendency for a negative correlation with fecal score during d 7–14 (r = −0.500; *p* < 0.10). Serum D-lactate levels on d 0 post-weaning were positively correlated (r = 0.826; *p* = 0.01) with WW in barrows but not in gilts. The level of serum malondialdehyde in high-WW pigs on d 7 tended to be less (*p* = 0.07) than that in low-WW pigs. Fecal acetate, propionate, butyrate, and total short-chain fatty acid concentrations were not affected by WW at d 0, 14, and 28 post-weaningg. Weaning weight was positively associated with the overall growth performance of nursery pigs with more pronounced effect in gilts while no interactions between WW and sex were observed in gut permeability, antioxidant status, and fecal short-chain fatty acid concentrations, implying that sex-specific nutritional strategies and precision weaning management are warranted.

## Simple Summary

The weight of piglets at weaning is crucial for their growth and health. In this study, we evaluated how weaning weight affects the growth and health status in nursery barrows and gilts. Pigs with a higher weaning weight consumed more feed and grew faster throughout the nursery period. Furthermore, pigs that achieved greater growth and feed intake during the first week of post-weaning demonstrated even better overall performance in the following weeks. These findings were particularly noticeable in gilts compared with barrows. Additionally, heavier piglets had an improved blood antioxidant status, although this did not differ between sexes. In contrast, gut permeability and the production of most short-chain fatty acids were not affected by weaning weight. In conclusion, heavier pigs at weaning, especially gilts, are more resilient to weaning stress, allowing them to maintain a greater growth rate and heavier body weight in nursery stage.

## 1. Introduction

Weaning is a critical phase in swine production, characterized by abrupt nutritional, environmental, and social changes that impose substantial stress on piglets. The shift from milk to solid feed challenges the immature digestive and immune systems, often resulting in intestinal inflammation, oxidative imbalance, and transient growth depression collectively known as post-weaning stress [1,2]. Such stress impairs gut morphology and barrier integrity, suppresses digestive enzyme activity, and increases inflammatory responses, leading to diarrhea, reduced feed intake, and poor growth [1,3]. 

The response to weaning stress can vary markedly among individuals, and one factor contributing to this variation is weaning weight (WW). The WW reflects the cumulative effects of prenatal development, maternal nutrition, and suckling growth [4,5], and serves as a useful indicator of overall vitality and resilience [6,7]. Kwon et al. [8] reported that piglets with high WW adapt more efficiently after weaning, showing greater early nursery growth rate and stronger immune competence and gut integrity compared with those with low WW, exhibiting enhanced antioxidant capacity and gut integrity during the first 2 weeks post-weaning. Similar findings have been consistently reported in previous studies [7,9].

In addition to WW, sex may also contribute to individual variation in post-weaning adaptation. It has been reported that gilts show faster intestinal recovery and greater digestive enzyme activity after weaning than barrows during the nursery [9,10]. These physiological differences suggest that gilts may cope with weaning-related stress more efficiently than barrows. Although differences in WW reflect variation in physiological maturity and stress sensitivity, how sex-related physiological differences affect the growth and health of pigs during the early nursery period, particularly in relation to WW, is still not fully understood. Given the differences in hormonal and immune responses between male and female pigs to stress conditions [11,12], different WW may show interaction with sex on piglets’ post-weaning performance and health markers.

Previous studies have focused either on the first 1–2 weeks after weaning or primarily on growth performance [8,10,13], providing limited information into how physiological indicators, such as antioxidant status, immune function, and gut health, change over the entire early nursery period. In addition, few studies have evaluated how individual effects of WW and sex interact with physiological indicators during the early nursery period. Despite standardized management practice in commercial swine farms, variations in growth performance driven by these factors can hinder systematic herd management.

Therefore, the present study was conducted to evaluate the effects of pig WW and sex on growth performance, fecal score, blood antioxidant capacity, and fecal short-chain fatty acid (SCFA) concentrations during the early nursery period, and to examine their relationships.

## 2. Materials and Methods

The experimental protocol was reviewed and approved by the Institutional Animal Care and Use Committee of the University of Georgia, USA (#A2024 01-023-Y2-A0).

### 2.1. Animals, Experimental Design, Diets, and Housing

At weaning at 19.0 ± 1.35 d of age, a total of 64 pigs (32 barrows and 32 gilts; Camborough × PIC337) from eight sow litters were selected based on their WW and health status. Each sow litter contributed three to nine piglets to each WW category. All piglets were allotted to two WW categories: high-WW pigs with body weight (BW) greater than 5.5 kg (average 6.30 ± 0.53 kg, 5.6 to 7.2 kg; average birth weight = 1.74 ± 0.30 kg) and low-WW pigs with BW less than 5.5 kg (average 4.85 ± 0.46 kg, 3.8 to 5.5 kg; average birth weight = 1.48 ± 0.31 kg). Each treatment had eight replicate pens (four pens for barrows and four pens for gilts) with four pigs per pen, balanced for littermate and initial BW, in a randomized complete block design. Prior to allocation, all piglets underwent health screening. Piglets exhibiting signs of severe emaciation, lethargy, or poor vitality were excluded to ensure the uniformity of the experimental animals.

Before weaning, routine piglet processing including an intramuscular injection of 200 mg iron dextran (UNIFERON^®^ 200, Pharmacosmos Inc., Watchung, NJ, USA), ear notching, tail docking, and castration was carried out by trained personnel following the standard operating procedures of the University of Georgia Swine Research Unit. All pigs were housed in nursery pens (2.0 m × 1.0 m) with plastic slatted flooring and had ad libitum access to water and feed in an environmentally controlled nursery room at the University of Georgia Swine Research Unit during the 28-day experimental period. Common corn-soybean meal-based diets were formulated to meet or exceed NRC [14] nutrient requirements. Pigs were fed a two-phase nursery program: Phase 1 (d 0–14) and Phase 2 (d 14–28; Table 1).

### 2.2. Data and Sample Collection

Individual body weight (BW) was recorded on the d 0, 7, 14, 21, and 28 early nursery period to calculate average daily gain (ADG). Feed disappearance per pen was measured at the same time to determine average daily feed intake (ADFI) and the gain-to-feed ratio (G:F). The fecal score was evaluated daily during the entire experimental period using a four-point scale (1 = normal, 2 = soft or slightly loose, 3 = moderate diarrhea, and 4 = watery, severe diarrhea) as described by Kwon et al. [8]. Individual pigs in each pen were observed, and fecal consistency was assessed accordingly.

For blood sampling, one pig in each pen (four pigs per treatment) was selected based on average BW within each pen. Approximately 10 mL of blood was collected via jugular venipuncture using serum separation tubes (Becton Dickinson, Franklin, NJ, USA) on d 0, 7, 14, 21, and 28 post-weaning. After clotting at room temperature for 30 min, serum samples were centrifuged at 2500× *g* for 30 min at 4 °C, and the obtained serum was stored at −80 °C until analysis. All serum samples were analyzed within 3 months of collection.

Fecal samples were collected from the same pigs used for blood sampling on d 0, 14, and 28. Fresh feces were obtained directly from the rectum into sterile cryogenic tubes, immediately snapped frozen in liquid nitrogen, and stored at −80 °C until further analysis.

### 2.3. Laboratory Analysis

Serum samples were analyzed for parameters for gut permeability and oxidative status. Diamine oxidase (DAO; 76714-548, AFG Bioscience, Northbrook, IL, USA) and D-lactate (EDLC-100, BioAssay Systems, Hayward, CA, USA) were measured as indicators of intestinal permeability using commercial kits. Malondialdehyde (MDA; 10009055), superoxide dismutase (SOD; 706002), and catalase (707002) were determined using commercial kits (Cayman Chemical Company, Ann Arbor, MI, USA) with a microplate spectrophotometer (MULTISKAN SkyHigh, Thermo Fisher Scientific, Waltham, MA, USA). All assays were performed in duplicate following the manufacturers’ instructions.

Fecal SCFA concentrations were determined following the method of Lourenco et al. [15] with minor modifications. Briefly, fecal samples were homogenized with distilled water (1:3, *w*/*v*) and centrifuged at 10,000× *g* for 10 min. One milliliter of the supernatant was mixed with 0.2 mL of 25% (*w*/*v*) metaphosphoric acid and stored at −20 °C overnight. After thawing, samples were centrifuged again, and the resulting supernatant was extracted with ethyl acetate (2:1, *v*/*v*). The upper phase (0.5 mL) was transferred into screw-cap vials for gas chromatograph analysis. Short-chain fatty acids were quantified using a gas chromatograph (GC-2010 Plus; Shimadzu, Kyoto, Japan) equipped with a flame ionization detector and a Zebron ZB-FFAP capillary column (30 m × 0.32 mm × 0.25 μm; Phenomenex, Torrance, CA, USA). The injection volume was 1 μL, whereby helium served as the carrier gas, and the oven temperature was programmed from 110 °C to 200 °C with injector and detector temperatures of 250 °C and 350 °C, respectively.

### 2.4. Statistical Analysis

All data obtained in the current study were analyzed by ANOVA using the PROC MIXED procedure of SAS (ver. 9.4; SAS Inst. Inc., Cary, NC, USA). All data were tested for normality by the Shapiro–Wilk test. The models included WW, sex, and their interaction as fixed effects with replicate as a random effect. The least square means were separated using the PDIFF option of SAS. Pearson correlation coefficients of WW or growth performance in the first week of post-weaning with post-weaning growth performance and fecal score were determined separately in barrows and gilts using PROC CORR of SAS. Pearson correlation coefficients between WW and serum parameters or fecal SCFA concentrations were determined regardless of sex but determined separately in barrows and gilts if the interaction between WW and sex was significant. A pen was used as an experimental unit for growth performance and fecal score while an individual pig was used as an experimental unit for serum parameters and fecal SCFA production. Statistical differences were established at *p* ≤ 0.05 and tendencies were established at 0.05 < *p* ≤ 0.10.

## 3. Results

### 3.1. Growth Performance

During the entire experimental period, high-WW pigs showed greater growth performance than low-WW pigs (Table 2). The BW in high-WW pigs was greater (*p* < 0.01) than that in low-WW pigs. High-WW pigs showed greater ADG during d 7–14 (tendency *p* = 0.08) and d 14–21 (*p* = 0.01) than low-WW pigs, resulting in greater ADG during d 0–14 (tendency *p* = 0.06), d 14–28 (*p* < 0.01), and the overall period (*p* < 0.01). High-WW pigs tended to show greater ADFI (*p* < 0.10) during d 7–14 and d 21–28 than low-WW pigs, resulting in greater ADFI during d 0–14, d 14–28, and the overall period (tendency *p* < 0.10), but no difference was observed in G:F between high-WW and low-WW pigs. 

Interactions between WW and sex were also observed on growth performance during post-weaning period. Weaning weight and sex had interactive effects on BW on d 21 (*p* = 0.02) and d 7, 14, and 28 (tendency *p* < 0.10). The ADG was affected by the interaction between WW and sex during d 0–7 (*p* = 0.03), d 14–21, d 0–14, and the overall period (tendency *p* < 0.10). The G:F was affected by the interaction between WW and sex during d 0–7 (*p* = 0.01), d 7–14 (*p* = 0.04), d 14–21 (tendency *p* = 0.08), d 0–14 (*p* < 0.01), and d 14–28 (tendency *p* = 0.06), and the overall period (*p* = 0.02). 

Weaning weight and the first week of post-weaning performance showed correlations with the subsequent performance throughout the experimental period depending on sex (Table 3). Weaning weight was positively correlated (*p* < 0.05) with BW throughout the experimental period but not correlated with ADG, ADFI, and G:F during d 0–7 in barrows and gilts. In barrows, WW tended to be negatively correlated (*p* < 0.10) with G:F during d 7–14, d 0–14, and the overall period but not correlated with ADG and ADFI throughout the experimental period. In contrast, in gilts, WW was positively correlated (*p* < 0.05) with ADG and ADFI during d 7–14, d 14–21, d 0–14, d 14–28, and the overall period. The ADG during d 0–7 was not correlated with BW throughout the experimental period in barrows but positively correlated (*p* < 0.10) in gilts. Growth performance of gilts during d 0–7 was positively correlated with ADG, ADFI, and G:F during d 7–14 (*p* < 0.05), d 0–14 (*p* < 0.05), and the overall period (*p* < 0.10) but not in barrows. 

### 3.2. Fecal Score

Postweaning fecal scores were not affected by WW, sex, or their interaction throughout the experimental period (Table 4). Weaning weight showed a tendency for a negative correlation with fecal score during d 7–14 (r = −0.500; *p* < 0.10) whereas no correlation between fecal score and growth performance in the first week of post-weaning was observed (Table 5).

### 3.3. Serum Parameters

At weaning, serum DAO levels in high-WW pigs tended to be greater (*p* = 0.09) than that in low-WW pigs, but no interaction between WW and sex on serum DAO was observed (Table 6). The serum D-lactate levels at weaning in high-WW pigs tended to be greater (*p* = 0.08) than that in low-WW pigs. The serum D-lactate level at weaning was affected by the interaction between WW and sex, showing a positive correlation (r = 0.826; *p* = 0.01) between serum D-lactate levels and WW in barrows but not in gilts. Gilts had greater (*p* < 0.05) serum D-lactate levels than barrows at d 21 post-weaning. 

Serum MDA levels in high-WW pigs tended to be less (*p* = 0.07) than those in low-WW pigs on d 7 but greater (*p* = 0.07) on 28 (Table 7). Serum catalase concentrations tended to be affected by the interaction between WW and sex (*p* = 0.09), but sex-specific relationships between serum catalase level and WW were not observed in Pearson correlation analysis.

In Pearson correlation analysis, WW was negatively correlated (r = −0.526; *p* < 0.10) with serum MDA level on d 7 regardless of sex (Table 8). Weaning weight was positively correlated with serum SOD level on d 21 (r = 0.581; *p* < 0.05) and 28 (r = 0.530; *p* < 0.05).

### 3.4. Fecal SCFA Concentrations and Composition

At weaning, fecal isobutyrate concentrations tended to be influenced by the interaction between WW and sex (*p* = 0.10; Table 9). On d 14, fecal isobutyrate concentrations in high-WW pigs tended to be less (*p* = 0.07) than that in low-WW pigs. On d 28, fecal valerate concentrations in gilts tended to be greater (*p* = 0.10) than that in barrows.

At weaning, fecal isobutyrate composition as % of total SCFA was affected (*p* = 0.02) by the interaction between WW and sex, showing the tendency for a negative correlation (r = −0.667; *p* = 0.07) with WW in barrows, but no correlation in gilts (Table 10). On d 14, fecal valerate composition in low-WW pigs tended to be greater (*p* = 0.07) than that in high-WW pigs. On d 28, fecal valerate composition in gilts tended to be greater (*p* = 0.06) than that in barrows.

Weaning weight was negatively correlated with concentrations of fecal isobutyrate (r = −0.497; *p* < 0.10), butyrate (r = −0.516; *p* < 0.05), and isovalerate (r = −0.524; *p* < 0.05) on d 28 (Table 11). Weaning weight tended to be positively correlated (r = 0.448; *p* < 0.10) with fecal acetate composition as % of total SCFA at weaning but was negatively correlated with fecal butyrate (r = −0.653; *p* < 0.01) and valerate composition (r = −0.513; *p* < 0.05) on d 28.

## 4. Discussion

Weaning is one of the most challenging issues during a pig’s entire life due to weaning stress, which is critical in post-weaning performance. One of the main factors affecting post-weaning growth is WW as pigs with heavy WW can overcome weaning stress more quickly compared with low-WW pigs [8,10,16]. However, the effect of WW may depend on the sex of the piglets, given the physiological differences between barrows and gilts [11,12,14]. In previous studies, gilts and barrows showed different responses such as circulating hormones including glucagon, triiodothyronine, and cortisol, muscle injury, and immune cell populations under stress condition [11,12], indicating that sex-specific resistance to stress exists. However, limited data are available on how WW and sex interact to influence post-weaning growth performance and health markers in nursery pigs. 

In the present experiment, high-WW pigs maintained a heavier weight until the end of the experimental period, which agrees with previous studies [8,16,17]. The results are due to the greater growth rate in high-WW pigs compared with low-WW pigs in every week except for the first week of post-weaning. The lack of difference in growth rate in the first week of post-weaning between high-WW and low-WW pigs observed in the present work has been consistently reported in the previous studies [8,13,18], indicating that weaning stress compromises the growth of all piglets regardless of WW immediately after weaning. The greater overall post-weaning growth rate in high-WW pigs resulted from greater overall feed intake throughout the entire nursery period while there was no difference in feed efficiency. This is consistent with previous findings that high-WW pigs showed greater weight gain during 20–21post-weaning because of increased feed intake with similar feed efficiency compared with low-WW pigs [7,18]. This indicates that feed consumption would be more important than improved feed efficiency in post-weaning period although the quality of diet (e.g., digestibility, complexity, fiber, and protein levels) can significantly influence the latter [19,20]. Low feed consumption contributes to the delayed development of the gastrointestinal tract and immune functions [9,10], and, consequently, growth retardation and higher mortality as observed in previous experiments [6,7]. 

In the present experiment, an interaction between WW and sex was observed on BW at d 7, 14, 21, and 28 post-weaning, which was consistent with the higher correlation coefficients between WW and BW observed every week in gilts compared with barrows. This result stemmed from the fact that the ADG in high-WW gilts was greater than that in low-WW gilts whereas barrows had relatively similar ADG regardless of WW, which consequently resulted in positive correlations between WW and post-weaning ADG in gilts, but not in barrows. This suggests that WW influences post-weaning growth performance more noticeably in gilts, particularly in high-WW gilts showing prominent weight gain and feed efficiency compared to barrows. The superior capacity of gilts to endure stress has been reported in a previous study, where gilts challenged with lipopolysaccharide exhibited lower serum norepinephrine concentrations despite similar serum cortisol levels [21], indicating that gilts possess lower stress sensitivity under challenging conditions. In addition, because serum pro-inflammatory cytokine levels such as tumor necrosis factor-α and interleukin-6 in gilts peaked rapidly and immediately returned to baseline levels after the challenge in the previous study [21], it possibly demonstrates that gilts possess a more efficient regulatory mechanism against acute inflammation caused by stressful condition than barrows. Similarly, in rats, females initially showed robust corticosterone secretion under restraint stress but displayed a faster recovery of plasma corticosterone levels back to baseline compared to males, along with a more active serotonin metabolism in the brain [22]. In contrast to the present findings, no interactive effect between WW and sex was observed on weight gain and feed efficiency in pigs in a previous experiment [18]. This inconsistency may be attributed to differences in WW and weaning age (5.6 kg weaned at d 19 in the present study vs. 6.6 kg weaned at d 24 in the previous study) [18] as both WW and weaning age influence post-weaning organ development and consequently growth performance [10,18]. In a previous experiment, the activities of maltase and glucoamylase in the small intestine and pancreatic amylase were greater in pigs weaned at 28 d of age than in those at 14 d of age [10]. Additionally, differences in pig breeds might have also confounded the effects of WW and sex between the present study (Camborough × PIC337) and the previous one (Large White × Landrace) [18] as growth rate and body composition are highly dependent on pig breed [23,24]. Particularly, the variation in maturity in the early post-weaning period among different breeds could result in difference in overall growth performance [24]. Given that few studies have investigated the interactive effect of WW and sex on post-weaning growth performance in pigs, further research is warranted to clarify these relationships.

In the correlation analysis, no relationship between WW and first-week growth performance was observed, which is consistent with the previous literature [8,13], suggesting that weaning remains a substantial stressor regardless of WW. However, weight gain, feed intake, and feed efficiency in the first week were positively correlated with those in the subsequent nursery period, suggesting that improving weight gain and feed intake during the first week can enhance growth performance throughout the overall nursery period. Faccin et al. [13] also observed a positive correlation between first-week ADG and the ADG or BW of the subsequent nursery period. In the present study, the effects of first-week growth performance were observed more prominently in gilts than barrows. However, limited information is currently available to explain the sex-specific influence.

It is noteworthy that first-week ADG and ADFI were positively correlated with feed efficiency on several time points in the present experiment, which aligns with the findings of Fabà et al. [25]. This indicates that feed intake and growth during the first week of post-weaning are critical factors for the development of the gastrointestinal tract and physiological functions, thereby improving the efficiency of protein and lipid deposition throughout the nursery period [26]. Supporting these findings, a previous study reported that nursery pigs with higher feed intake exhibited heavier small and large intestines and greater villus heights in three sections of the small intestine on d 5 post-weaning [27]. Fabà et al. [25] also observed increased jejunal villus heights and heavier weights of the small and large intestines on d 6 post-weaning in pigs fed higher amounts of feed for 3 d after weaning, which improved both growth rate and feed efficiency. Therefore, increasing weight gain and feed intake in the first week post-weaning could act as a driving force for rapidly overcoming weaning stress and facilitating the recovery of underdeveloped organs [8,13]. However, in the present experiment, these relationships were more prominent in gilts compared to barrows, which could be related to the greater enzyme activity and nutrient absorption capacity in gilts under a similar feed intake to boars [10]. 

In the present experiment, a negative correlation was observed between WW and fecal score during d 7–14, but not during d 0–7. This is likely because weaning stress, including the transition from milk to solid feed and changes in environmental conditions, impaired intestinal barrier function and induced diarrhea regardless of piglet WW during the first week [2,26]. This aligns with the lack of correlation between WW and growth performance during the first week in both barrows and gilts. After the first week of post-weaning, high-WW pigs appeared to overcome stress more rapidly than low-WW pigs, resulting in improved fecal consistency with increasing WW in d 7–14, resulting in a negative correlation between WW and fecal score during d 7–14. Subsequently, the fecal scores of low-WW pigs also appeared to stabilize as the pigs passed the most stressful transition period.

Serum DAO concentrations were determined in the present experiment to evaluate the effect of WW on gut permeability in nursery pigs [28]. A tendency for increased serum DAO concentration in high-WW pigs was observed compared to low-WW pigs at weaning but not observed in the subsequent periods. The increased DAO concentration in high-WW pigs immediately after weaning (d 0) may indicate greater DAO synthesis due to a relatively more developed intestinal barrier function than that of low-WW pigs. Postnatal and post-weaning periods are characterized by rapid developmental changes in the gastrointestinal tract, during which cell proliferation increases several-fold, enhancing the activity of polyamine metabolism enzymes such as DAO and ornithine decarboxylase [29]. This may partially contribute to the improved feed intake and weight gain for first 7 d post-weaning in high-WW pigs compared to low-WW pigs in the present study. In addition, a previous study also observed a gradual increase in plasma DAO activity in nursery pigs over time [30], indicating that blood DAO activity increases in tandem with intestinal development as pigs grow. By the same token, the nursery pigs used in the present study showed numerically higher serum DAO concentrations on d 2 post-weaning compared to those at weaning.

However, the lack of difference in serum DAO concentrations between high- and low-WW pigs during the post-weaning period observed in the present experiment contradicts previous observations where high-WW and low-WW pigs showed no difference in plasma DAO concentration on d 7 post-weaning (36.77 vs. 34.87 ng/mL, respectively), but increased concentrations were observed in low-WW pigs on d 1 post-weaning (27.42 vs. 37.15 ng/mL, respectively) [8]. Generally, increased blood DAO concentration is interpreted as elevated gut permeability resulting from impaired gut barrier function and the subsequent leakage of DAO from intestinal epithelial cells into the peripheral blood [28,30,31]. In this context, the findings by Kwon et al. [8] indicate that weaning stress compromised gut barrier function in all piglets during the first week, but high-WW pigs recovered intestinal barrier integrity more rapidly than low-WW pigs by d 14 post-weaning. Considering the lower average serum DAO concentrations on d 7 (22.92 vs. 34.87 ng/mL) and d 14 (20.48 vs. 37.15 ng/mL) in low-WW pigs in the present experiment compared to those in Kwon et al. [8] despite similar BW, it suggests that the intestinal barrier of the piglets in the present experiment was not sufficiently compromised to alter serum DAO concentration.

D-lactate is also a maker of gut permeability but distinct from DAO in that D-lactate is produced by colonic anaerobic microbial fermentation and leaks from the intestinal lumen into the systemic circulation under impaired intestinal conditions [32]. In the present experiment, serum D-lactate levels tended to increase with increasing WW, but this increase was observed only in barrows and not in gilts at weaning, demonstrating a positive correlation between WW and D-lactate levels exclusively in barrows. Similarly to the serum DAO concentrations mentioned above, the increased serum D-lactate levels with increasing WW immediately after weaning in barrows may also be attributed to the relatively mature intestines. However, the data regarding why WW exerts a greater influence on serum D-lactate levels in barrows than in gilts are limited. Potential factors affecting blood D-lactate levels sex-specifically at weaning might be related to differences in gut microbial composition, specifically those microbes responsible for D-lactate production [32], or to gut function development depending on both WW and sex. 

Previous studies reported that blood D-lactate levels increase by leaking into the bloodstream due to elevated intestinal permeability under stressful conditions [31,33]. However, the present finding that gilts showed greater D-lactate levels than barrows on d 21 may not necessarily be attributed to a leaky gut induced by weaning stress, given that high-WW gilts exhibited the greatest weight gain, feed intake, and feed efficiency throughout the entire nursery period. Because limited data are available regarding the effects of WW and sex on blood D-lactate levels, further research is warranted to accumulate data on how WW and the physiological differences between barrows and gilts influence gut permeability in nursery pigs.

Serum MDA is a marker of lipid peroxidation status and has been reported in previous studies to increase under challenging conditions, reflecting increased oxidative stress [34,35]. In the present work, serum MDA levels at weaning did not differ among treatment groups, but low-WW pigs tended to have higher serum MDA levels than high-WW pigs on d 7 post-weaning. This aligns with the negative relationship between WW and serum MDA on d 7 in the current correlation analysis, likely indicating that low-WW pigs are more susceptible to oxidative injury than high-WW pigs during the first week of post-weaning. Luo et al. [35] also observed that hepatic MDA levels did not differ among non-weaning and weaning groups at weaning but showed differences on d 4 and 7 post-weaning with increased MDA levels in the weaning group, indicating a postponed response of MDA to weaning stress. The increased MDA levels during the first week post-weaning observed in the present work may be partially related to low feed intake and weight gain in low-WW pigs compared with high-WW pigs.

The reason for the tendency toward higher serum MDA levels in high-WW pigs on d 28 post-weaning remains unclear. In fact, the effects of lipid peroxidation and oxidative stress on blood MDA levels remain controversial in the literature. Whereas oxidative stress conditions have been reported to increase blood MDA levels [34,35], other studies observed no difference in the oxidative stress-challenged group [36] or the low-WW group [8,17]. Moreover, Silva–Guillen et al. [37] reported decreased serum MDA levels in pigs consuming peroxidized oil compared to the control group. Given that few studies have investigated the effects of WW on blood MDA levels, further accumulated data are needed to clarify the effects of WW on oxidative status, particularly MDA levels, in pigs.

Superoxide dismutase is an enzymatic antioxidant that scavenges reactive oxygen species in the body [35,37]. In the present experiment, WW was not correlated with serum SOD concentrations at d 0, 7, and 14 post-weaning but showed a positive correlation at d 21 and 28. This partially agrees with Kwon et al. [8] who observed that high-WW pigs had similar plasma SOD concentrations to low-WW pigs at d 7 post-weaning but greater at d 1 post-weaning. These findings indicate that antioxidant capacity is suppressed by weaning stress during the early post-weaning period in all piglets regardless of WW, but high-WW pigs appear to recover the antioxidant capacity after 2 to 3 weeks post-weaning whereas low-WW pigs may require more time to restore it. The enhanced antioxidant capacity likely contributed to the greater weight gain and feed intake observed in high-WW pigs in the present study.

In the present experiment, fecal isobutyrate concentration and composition (% of total SCFA) increased with increasing WW in gilts at weaning prior to the consumption of solid feed. This result may be partially explained by higher milk consumption during the suckling period in high-WW gilts compared with low-WW gilts as indicated by their heavier BW at weaning. Because isobutyrate is produced through the fermentation of branched-chain amino acids by gut microbiota [38], an increased amount of undigested protein entering the hindgut resulting from higher branched-chain amino acid consumption via milk potentially leads to increased isobutyrate production. Interestingly, a decrease in isobutyrate concentration and composition (% of total SCFA) at weaning with increasing WW was observed in barrows, showing a tendency toward a negative correlation between WW and isobutyrate composition (% total SCFA). This contradicts no correlation observed in gilts in the present study. It is possible that differences in gut microbiota composition between barrows and gilts affected protein fermentation and metabolite production [39,40]. Conversely, a previous study observed no difference in isobutyrate production between male and female pigs at 5 kg or 7 kg BW [40]. 

In contrast to the results at weaning, isobutyrate concentration on d 14 tended to be higher in low-WW pigs than in high-WW pigs likely because low-WW pigs had a lower capacity to digest protein in solid feed, causing more undigested protein to enter the hindgut. This leads to increased protein fermentation, specifically of branched-chain amino acids, by microbiome [38]. However, isobutyrate concentration did not differ between high-WW and low-WW pigs on d 28, indicating that the piglets had adapted to the solid feed and environmental changes, and their gastrointestinal capacity had stabilized over time. Similarly, Yao et al. [40] observed higher colonic isobutyrate production in 11 kg gilts compared with barrows, but this difference disappeared as the pigs reached a BW of 20 kg or 25 kg.

Along with the increase in total SCFA production, valerate production also increased in all piglets by d 28 compared to d 0 and 14. However, valerate concentration tended to be higher in gilts than in barrows with a greater composition (% of total SCFA) on d 28. Valerate is produced not only through the fermentation of amino acids such as proline, but also via the conversion of acetate and propionate to acetyl-CoA and propionyl-CoA, respectively, followed by their condensation This may suggest that valerate production involves a complex fermentation process [38]. Furthermore, the observed differences in fecal valerate concentrations may be attributed to variations in the abundance of specific valerate-producing microbial genera such as *Oscillibacter* between barrows and gilts [40]. In contrast, the production of predominant SCFA such as acetate and butyrate, which are primarily influenced by energy metabolism and the type of carbohydrates consumed, remains relatively consistent regardless of sex [19,40]. Therefore, the present findings potentially imply that intestinal development and microbial composition become distinct between barrows and gilts by d 28.

In the present correlation study, however, no sex-specific relationship between WW and SCFA production was observed except for isobutyrate composition. The negative correlations between WW and the production of isobutyrate, butyrate, and isovalerate on d 28 regardless of sex may be attributed to the immature gastrointestinal tract of lower-WW pigs with a lower capacity to digest and absorb dietary nutrients, allowing more undigested nutrients to enter the hindgut and undergo microbial fermentation. Previous studies have reported higher SCFA production in the large intestine of pigs fed less digestible diets [41,42]. This suggests that elevated SCFA production can serve as a marker not only of beneficial influences on gut health but also of the quantity of undigested nutrients entering the hindgut. The latter appears to be the case of the present observations, partially supporting the reduced weight gain and several health markers in low-WW pigs. However, total SCFA concentration was not affected by WW or sex throughout the experimental period. This is because the branched-chain SCFA produced by protein fermentation such as isobutyrate and valerate is produced in much smaller quantities compared to predominant SCFA like acetate and propionate, thereby making up only a minor proportion of the total SCFA.

## 5. Conclusions

In the present experiment, high-WW pigs exhibited greater feed intake and weight gain, resulting in a heavier BW compared to low-WW pigs throughout the 28 d nursery period without affecting feed efficiency, suggesting that increased feed intake is the primary factor for improving post-weaning growth. Positive correlations between WW and growth performance were observed in gilts, but not necessarily in barrows. Additionally, early post-weaning performance was sex-specifically associated with subsequent growth, but this still indicates that overall nursery performance is largely determined by early-period growth along with the pig WW. Several health markers collectively demonstrated that high-WW pigs possess a greater capacity to rapidly overcome weaning stress. However, further studies are warranted to investigate the interactive effects of WW and sex on hormonal responses and inflammatory status under weaning stress. Such research will provide a clearer explanation for the current growth performance and health status outcomes, thereby elucidating the underlying physiological mechanisms.

## Figures and Tables

**Table 1:** Diet formulation and calculated chemical composition.

Ingredients, %	d 0–14 Postweaning (Phase 1)	d 14–28 Postweaning (Phase 2)
Corn	26.71	43.75
Soybean meal (48% crude protein)	14.64	27.10
Dried whey	15.00	5.00
Oats	10.00	5.00
HP300 ^1^	10.00	5.00
Lactose	10.00	5.00
Fish meal	5.00	3.00
Animal plasma	3.00	-
Soybean oil	2.96	2.90
L-Lysine-HCl	0.30	0.30
DL-Methionine	0.11	0.11
L-Threonine	0.06	0.06
Dicalcium phosphate	0.47	0.91
Limestone	0.85	0.85
Salt	0.50	0.50
Trace mineral mix ^2^	0.15	0.15
Vitamin mix ^3^	0.25	0.25
Total	100.00	100.00
Calculated chemical composition		
Metabolizable energy, kcal/kg	3444	3415
Crude protein, %	23.68	22.72
SID lysine, %	1.51	1.34
SID methionine + cysteine, %	0.78	0.71
Total Ca, %	0.83	0.79
STTD P, %	0.42	0.37

SID = standardized ileal digestible; STTD = standardized total tract digestible. ^1^ Hamlet Protein, Findlay, OH, USA. ^2^ Trace mineral premix supplied the following per kilogram of diet: 33 mg of Mn as manganous oxide, 110 mg of Fe as ferrous sulfate, 110 mg of Zn as zinc sulfate, 16.5 mg of Cu as copper sulfate, 0.3 mg of I as Ca iodate, and 0.3 mg of Se as sodium selenite. ^3^ Vitamin premix supplied the following per kilogram of diet: 11,000 IU of vitamin A, 1600 IU of vitamin D_3_, 99 IU of vitamin E, 4.4 mg of vitamin K, 55 µg of vitamin B_12_, 9.9 mg of riboflavin, 31.9 mg of pantothenic acid, 55 mg of niacin, 0.9 mg of folic acid, 3.9 mg of vitamin B_6_, 3.1 mg of thiamin, 0.3 mg of biotin, and 600 mg of choline chloride.

**Table 2:** Effects of weaning weight (WW) and sex on post-weaning growth performance in nursery pigs ^1,2^.

Item	High WW		Low WW		SEM	*p*-Value		
	Barrow	Gilt	Barrow	Gilt		WW	Sex	WW × Sex
Body weight, kg								
d 0	6.26	6.32	4.84	4.85	0.26	<0.01	0.48	0.56
d 7	6.61	6.92	5.30	5.22	0.31	<0.01	0.25	0.07
d 14	8.65	9.26	7.21	6.80	0.47	<0.01	0.69	0.07
d 21	11.88	12.87	10.19	9.52	0.60	<0.01	0.59	0.02
d 28	14.60	15.71	12.56	11.95	0.72	<0.01	0.53	0.06
ADG, kg/d								
d 0–7	0.051	0.086	0.066	0.053	0.016	0.35	0.24	0.03
d 7–14	0.285	0.334	0.273	0.225	0.033	0.08	0.98	0.15
d 14–21	0.455	0.516	0.425	0.388	0.028	0.01	0.63	0.07
d 21–28	0.382	0.405	0.339	0.348	0.032	0.12	0.57	0.81
d 0–14 (Phase I)	0.171	0.210	0.169	0.139	0.022	0.06	0.77	0.06
d 14–28 (Phase II)	0.422	0.461	0.382	0.368	0.023	<0.01	0.49	0.17
d 0–28 (overall)	0.297	0.335	0.276	0.254	0.019	<0.01	0.56	0.06
ADFI, kg/d								
d 0–7	0.144	0.158	0.130	0.138	0.014	0.11	0.25	0.77
d 7–14	0.372	0.402	0.321	0.288	0.039	0.06	0.97	0.43
d 14–21	0.826	0.801	0.734	0.704	0.061	0.14	0.64	0.97
d 21–28	0.941	0.835	0.739	0.753	0.075	0.09	0.56	0.45
d 0–14 (Phase I)	0.260	0.280	0.225	0.213	0.024	0.05	0.88	0.49
d 14–28 (Phase II)	0.884	0.818	0.736	0.729	0.060	0.07	0.54	0.63
d 0–28 (overall)	0.567	0.549	0.481	0.471	0.037	0.06	0.71	0.91
G:F								
d 0–7	0.332	0.534	0.495	0.360	0.09	0.92	0.55	0.01
d 7–14	0.751	0.828	0.854	0.782	0.03	0.38	0.93	0.04
d 14–21	0.558	0.645	0.583	0.553	0.03	0.28	0.37	0.08
d 21–28	0.407	0.482	0.462	0.465	0.03	0.53	0.21	0.24
d 0–14 (Phase I)	0.646	0.746	0.753	0.639	0.04	1.00	0.82	<0.01
d 14–28 (Phase II)	0.475	0.564	0.519	0.507	0.02	0.78	0.14	0.06
d 0–28 (overall)	0.512	0.611	0.573	0.538	0.02	0.81	0.19	0.02

SEM = standard error of the means; ADG = average daily gain; ADFI = average daily feed intake; G:F = gain-to-feed ratio. ^1^ *n* = 4 replicate pens consisting of 4 pigs per pen. ^2^ Treatments: high-WW pigs with body weight over 5.5 kg (average 6.30 ± 0.53 kg) and low-WW pigs with body weight less than 5.5 kg (average 4.85 ± 0.46 kg).

**Table 3:** Pearson correlation coefficients between weaning weight (WW) or first-week post-weaning growth performance and nursery growth performance in barrows and gilts (n = 8).

Item	Barrow				Gilt			
	WW	d 0–7 ADG	d 0–7 ADFI	d 0–7 G:F	WW	d 0–7 ADG	d 0–7 ADFI	d 0–7 G:F
Body weight								
d 7	0.966 ***	0.131	0.435	−0.087	0.979 ***	0.620 *	0.573	0.579
d 14	0.879 ***	0.205	0.594	−0.090	0.932 ***	0.710 **	0.694 *	0.656 *
d 21	0.894 ***	0.055	0.377	−0.154	0.955 ***	0.644 *	0.645 *	0.602
d 28	0.817 **	−0.011	0.288	−0.191	0.939 ***	0.692 *	0.671 *	0.643 *
ADG								
d 0–7	−0.130	-	-	-	0.445	-	-	-
d 7–14	−0.072	0.229	0.515	−0.024	0.774 **	0.799 **	0.834 ***	0.725 **
d 14–21	0.462	−0.411	−0.454	−0.260	0.919 ***	0.406	0.453	0.401
d 21–28	0.377	−0.187	−0.037	−0.239	0.469	0.613 *	0.507	0.556
d 0–14 (Phase 1)	−0.121	0.682 *	0.771 **	0.442	0.712 **	0.891 ***	0.889 ***	0.827 **
d 14–28 (Phase 2)	0.443	−0.307	−0.238	−0.265	0.914 ***	0.631 *	0.604	0.595
d 0–28 (overall)	0.295	0.120	0.225	0.024	0.853 ***	0.794 **	0.779 **	0.742 **
ADFI								
d 0–7	0.238	0.754 *	-	-	0.417	0.900 ***	-	-
d 7–14	0.378	0.045	0.552	−0.329	0.735 **	0.721 **	0.783 **	0.644 *
d 14–21	0.417	−0.590	−0.548	−0.472	0.735 **	0.397	0.543	0.319
d 21–28	0.627	−0.383	0.026	−0.615	0.536	0.723 **	0.732 **	0.641 *
d 0–14 (Phase 1)	0.369	0.329	0.795 *	−0.078	0.698 **	0.782 **	0.854 ***	0.698 **
d 14–28 (Phase 2)	0.597	−0.518	−0.233	−0.614	0.716 **	0.642 *	0.726 **	0.552
d 0–28 (overall)	0.625	−0.393	−0.029	−0.573	0.714 **	0.703 **	0.783 **	0.615 *
G:F								
d 0–7	−0.322	0.901 ***	0.410	-	0.407	0.970 ***	0.799 **	-
d 7–14	−0.734 *	0.196	−0.190	0.432	0.459	0.828 **	0.740 **	0.842 ***
d 14–21	−0.307	0.708 *	0.620	0.607	0.665 *	0.231	0.175	0.301
d 21–28	−0.55	0.282	−0.146	0.574	0.093	0.131	−0.062	0.137
d 0–14 (Phase 1)	−0.676 *	0.512	−0.021	0.747 *	0.522	0.956 ***	0.818 **	0.981 ***
d 14–28 (Phase 2)	−0.613	0.461	0.072	0.681 *	0.593	0.229	0.109	0.285
d 0–28 (overall)	−0.667 *	0.563	0.151	0.750 *	0.719 **	0.650 *	0.510	0.688 *

ADG = average daily gain; ADFI = average daily feed intake; G:F = gain-to-feed ratio. * *p* < 0.10; ** *p* < 0.05; *** *p* < 0.01.

**Table 4:** Effects of weaning weight (WW) and sex on fecal score in nursery pigs ^1,2,3^.

Item	High WW		Low WW		SEM	*p*-Value		
	Barrow	Gilt	Barrow	Gilt		WW	Sex	WW × Sex
Fecal score								
d 0–7	1.52	1.46	1.34	1.41	0.08	0.20	0.95	0.46
d 7–14	1.40	1.32	1.48	1.57	0.13	0.21	0.96	0.52
d 14–21	1.29	1.38	1.27	1.38	0.12	0.94	0.42	0.94
d 21–28	1.09	1.29	1.09	1.14	0.10	0.46	0.19	0.44
d 0–14	1.47	1.39	1.41	1.49	0.10	0.83	0.98	0.43
d 14–28	1.19	1.33	1.18	1.26	0.08	0.61	0.20	0.71
d 0–28	1.31	1.36	1.29	1.38	0.07	0.99	0.35	0.84

SEM = standard error of the means. ^1^ *n* = 4 replicate pens consisting of 4 pigs per pen. ^2^ Treatments: high-WW pigs with body weight over 5.5 kg (average 6.30 ± 0.53 kg) and low-WW pigs with body weight less than 5.5 kg (average 4.85 ± 0.46 kg). ^3^ The fecal score was evaluated daily during the entire experimental period using a 4-point scale (1 = normal, 2 = soft or slightly loose, 3 = moderate diarrhea, and 4 = watery, severe diarrhea).

**Table 5:** Pearson correlation coefficients between weaning weight or first-week post-weaning growth performance and fecal score in nursery pigs (n = 16).

Item	Weaning Weight	d 0–7 ADG	d 0–7 ADFI	d 0–7 G:F
Fecal score				
d 0–7	0.252	0.026	0.151	−0.012
d 7–14	−0.500 *	−0.195	−0.136	−0.170
d 14–21	−0.156	−0.193	−0.025	−0.298
d 21–28	0.356	−0.066	0.160	−0.077
d 0–14	−0.245	−0.130	−0.027	−0.130
d 14–28	0.116	−0.173	0.083	−0.252
d 0–28	−0.093	−0.183	0.030	−0.227

ADG, average daily gain; ADFI, average daily feed intake; G:F, gain-to-feed ratio. * *p* < 0.10.

**Table 6:** Effects of weaning weight (WW) and sex on serum diamine oxidase and D-lactate levels in nursery pigs ^1,2^.

Item	High WW		Low WW		SEM	*p*-Value		
	Barrow	Gilt	Barrow	Gilt		WW	Sex	WW × Sex
Diamin oxidase, ng/mL								
d 0	29.11	26.91	25.39	26.05	1.81	0.09	0.55	0.26
d 7	26.44	20.54	23.01	22.82	1.78	0.75	0.12	0.14
d 14	24.00	19.71	19.75	21.21	2.06	0.46	0.45	0.14
d 21	23.69	22.77	22.93	24.15	2.20	0.86	0.94	0.56
d 28	28.93	31.04	28.33	27.47	1.39	0.11	0.61	0.23
D-lactate, mM								
d 0 ^3^	0.467	0.392	0.340	0.408	0.029	0.08	0.90	0.03
d 7	0.345	0.227	0.270	0.264	0.047	0.62	0.12	0.16
d 14	0.320	0.335	0.295	0.340	0.037	0.78	0.41	0.67
d 21	0.347	0.502	0.338	0.437	0.058	0.53	0.05	0.64
d 28	0.462	0.460	0.520	0.404	0.062	0.99	0.35	0.37

SEM = standard error of the means. ^1^ *n* = 4. ^2^ Treatments: high-WW pigs with body weight over 5.5 kg (average 6.30 ± 0.53 kg) and low-WW pigs with body weight less than 5.5 kg (average 4.85 ± 0.46 kg). ^3^ D-lactate on d 0 was positively correlated (r = 0.826; *p* = 0.01) with weaning weight of barrows (n = 8) but not correlated with weaning weight of gilts (n = 8).

**Table 7:** Effects of weaning weight (WW) and sex on serum malondialdehyde, superoxide dismutase, and catalase levels in nursery pigs ^1,2^.

Item	High WW		Low WW		SEM	*p*-Value		
	Barrow	Gilt	Barrow	Gilt		WW	Sex	WW × Sex
Malondialdehyde, µM								
d 0	10.53	10.99	14.45	10.83	1.38	0.20	0.27	0.16
d 7	6.89	6.63	7.64	7.76	0.49	0.07	0.87	0.68
d 14	7.54	7.17	6.56	7.12	0.36	0.21	0.82	0.26
d 21	5.96	5.73	5.82	5.68	0.47	0.81	0.63	0.90
d 28	4.27	4.28	4.10	3.88	0.15	0.07	0.47	0.43
Superoxide dismutase, U/mL								
d 0	5.62	6.33	6.15	6.98	0.50	0.25	0.14	0.91
d 7	7.20	5.98	5.86	5.23	0.71	0.18	0.23	0.67
d 14	7.64	6.97	7.72	7.86	0.40	0.25	0.52	0.33
d 21	8.32	7.59	7.49	8.04	0.43	0.61	0.81	0.12
d 28	9.61	9.73	8.53	7.85	0.99	0.15	0.77	0.68
Catalase, nmol/min/mL								
d 0	330	384	345	375	32	0.92	0.18	0.69
d 7	366	468	349	411	68	0.56	0.22	0.76
d 14	294	262	250	284	36	0.75	0.98	0.33
d 21	371	329	249	304	38	0.10	0.88	0.26
d 28	257	381	410	296	64	0.59	0.94	0.09

SEM = standard error of the means. ^1^ *n* = 4. ^2^ Treatments: high-WW pigs with body weight over 5.5 kg (average 6.30 ± 0.53 kg) and low-WW pigs with body weight less than 5.5 kg (average 4.85 ± 0.46 kg).

**Table 8:** Pearson correlation coefficients between weaning weight (WW) and serum parameters in nursery pigs (n = 16).

Item	Serum Parameter				
	d 0	d 7	d 14	d 21	d 28
WW vs. diamine oxidase	0.319	0.035	0.041	0.024	0.113
WW vs. D-lactate	0.420	0.182	0.090	0.311	−0.066
WW vs. malondialdehyde	−0.210	−0.526 *	0.102	0.392	0.057
WW vs. superoxide dismutase	−0.159	0.473	−0.048	0.581 **	0.530 **
WW vs. catalase	−0.189	0.167	−0.143	0.210	−0.060

* *p* < 0.10. ** *p* < 0.05.

**Table 9:** Effects of weaning weight (WW) and sex on fecal short-chain fatty acid (SCFA) concentrations (mM) in nursery pigs ^1,2^.

Item	High WW		Low WW		SEM	*p*-Value		
	Barrow	Gilt	Barrow	Gilt		WW	Sex	WW × Sex
d 0								
Acetate	33.06	42.42	33.13	31.51	7.59	0.49	0.62	0.48
Propionate	5.11	11.57	8.44	9.14	3.35	0.89	0.31	0.41
Isobutyrate	1.27	2.43	2.08	1.60	0.46	0.98	0.48	0.10
Butyrate	4.03	9.91	4.90	6.52	2.70	0.65	0.19	0.45
Isovalerate	2.52	4.96	3.78	3.59	0.93	0.95	0.25	0.18
Valerate	1.88	2.88	2.53	1.95	0.56	0.81	0.72	0.19
Total SCFA	48.89	74.68	55.84	54.35	13.85	0.64	0.40	0.34
d 14								
Acetate	68.91	73.20	64.41	66.41	6.99	0.44	0.66	0.87
Propionate	24.33	27.27	29.49	22.45	4.04	0.97	0.62	0.24
Isobutyrate	1.02	1.35	2.06	1.71	0.35	0.07	0.97	0.35
Butyrate	13.34	14.04	16.89	10.60	2.84	0.98	0.34	0.24
Isovalerate	1.77	1.82	3.24	2.42	0.62	0.12	0.54	0.50
Valerate	2.64	2.78	4.96	3.39	0.92	0.14	0.45	0.37
Total SCFA	112.27	120.66	121.78	107.66	12.75	0.89	0.83	0.39
d 28								
Acetate	63.68	55.24	50.13	55.72	5.77	0.22	0.78	0.19
Propionate	37.54	39.48	34.47	37.07	5.04	0.50	0.57	0.93
Isobutyrate	1.92	2.48	2.20	2.36	0.46	0.83	0.31	0.57
Butyrate	11.19	15.19	15.37	14.10	3.24	0.49	0.54	0.25
Isovalerate	2.96	4.06	3.42	3.94	0.83	0.76	0.16	0.61
Valerate	6.08	8.54	5.03	7.90	1.96	0.57	0.10	0.89
Total SCFA	124.20	126.06	111.41	123.59	15.38	0.45	0.49	0.61

SEM = standard error of the means ^1^ *n* = 4. ^2^ Treatments: high-WW pigs with body weight over 5.5 kg (average 6.30 ± 0.53 kg) and low-WW pigs with body weight less than 5.5 kg (average 4.85 ± 0.46 kg).

**Table 10:** Fecal short-chain fatty acid (SCFA) composition (% of total SCFA) of pigs with different weaning weight (WW) and sex ^1,2^.

Item	High WW		Low WW		SEM	*p*-Value		
	Barrow	Gilt	Barrow	Gilt		WW	Sex	WW × Sex
d 0								
Acetate	68.63	59.19	60.03	57.00	4.11	0.18	0.13	0.41
Propionate	10.70	14.65	14.74	12.98	2.78	0.68	0.70	0.32
Isobutyrate ^3^	2.61	3.33	3.91	2.90	0.31	0.19	0.65	0.02
Butyrate	8.68	11.79	9.35	15.01	3.13	0.50	0.15	0.66
Isovalerate	5.36	6.71	7.16	7.75	1.35	0.31	0.48	0.78
Valerate	4.03	4.33	4.82	4.36	1.16	0.71	0.94	0.73
d 14								
Acetate	61.83	60.70	54.03	62.36	3.22	0.36	0.28	0.17
Propionate	21.36	22.68	23.88	20.91	1.36	0.78	0.54	0.13
Isobutyrate	1.08	1.13	1.83	1.58	0.39	0.15	0.81	0.70
Butyrate	11.54	11.65	13.34	9.79	1.19	0.98	0.17	0.15
Isovalerate	1.85	1.53	2.96	2.25	0.72	0.22	0.49	0.79
Valerate	2.34	2.30	3.96	3.11	0.62	0.07	0.49	0.53
d 28								
Acetate	52.17	45.34	45.89	46.17	2.41	0.21	0.14	0.11
Propionate	30.63	31.43	30.99	30.63	1.56	0.89	0.89	0.72
Isobutyrate	1.48	2.08	1.88	1.93	0.28	0.64	0.24	0.33
Butyrate	8.65	11.39	13.95	11.65	1.43	0.05	0.86	0.07
Isovalerate	2.27	3.37	2.87	3.22	0.52	0.63	0.14	0.42
Valerate	4.79	6.39	4.44	6.39	1.00	0.84	0.06	0.84

SEM = standard error of the means ^1^ *n* = 4. ^2^ Treatments: high-WW pigs with body weight over 5.5 kg (average 6.30 ± 0.53 kg) and low-WW pigs with body weight less than 5.5 kg (average 4.85 ± 0.46 kg). ^3^ Fecal isobutyrate concentration on d 0 tended to be negatively correlated (r = −0.667; *p* = 0.07) with weaning weight of barrows (n = 8) but not correlated with weaning weight of gilts (n = 8).

**Table 11:** Pearson correlation coefficients between weaning weight (WW) and fecal short-chain fatty acid (SCFA) in nursery pigs (n = 16).

Item	Fecal SCFA		
	d 0	d 14	d 28
Fecal SCFA concentration (mM)			
WW vs. acetate	0.193	−0.054	−0.075
WW vs. propionate	−0.055	−0.169	−0.231
WW vs. isobutyrate	−0.082	−0.292	−0.497 *
WW vs. butyrate	−0.102	−0.181	−0.516 **
WW vs. isovalerate	−0.100	−0.244	−0.524 **
WW vs. valerate	−0.081	−0.325	−0.279
WW vs. total SCFA	0.067	−0.188	−0.323
Fecal SCFA composition (% of total SCFA)			
WW vs. acetate	0.448 *	0.197	−0.144
WW vs. propionate	−0.013	0.095	−0.409
WW vs. isobutyrate	−0.338	−0.262	−0.334
WW vs. butyrate	−0.361	0.143	−0.653 ***
WW vs. isovalerate	−0.333	−0.254	−0.398
WW vs. valerate	−0.224	0.174	−0.513 **

* *p* < 0.10; ** *p* < 0.05; *** *p* < 0.01.

## Data Availability

Upon reasonable request, the datasets of this study can be available from the corresponding author.

## Abbreviations

The following abbreviations are used in this manuscript:

ADFI	Average daily feed intake
ADG	Average daily gain
BW	Body weight
DAO	Diamine oxidase
G:F	Gain-to-feed ratio
MDA	Malondialdehyde
SCFA	Short-chain fatty acid
SOD	Superoxide dismutase
WW	Weaning weight
